# Microsatellite variation and allele frequency distribution for (TPOX) STRS locus in North Indian Muslim populations

**DOI:** 10.1186/1755-8166-7-S1-P108

**Published:** 2014-01-21

**Authors:** Ruqaiya Hussain, Mohammad Afzal

**Affiliations:** 1 Human Genetics and Toxicology Lab., Section of Genetics, Department of Zoology, Aligarh Muslim University, Aligarh, Uttar Pradesh, India

## Background

Autosomal short tandem repeats (STRs) have become the most informative molecular markers because they are highly polymorphic and multiallelic. The objective of the present work is to study the genotypic polymorphisms, allele frequencies and forensic parameters at highly polymorphic STR loci (TPOX) among four Muslim Populations of North India: Pathan, Ansari, Saifi and Mansoori.

## Materials and methods

The DNA was extracted from blood samples by using the standard phenol-chloroform extraction method and then amplified by PCR using specific primers for TPOX locus. The PCR products were separated by electrophoresis on denaturing 6% polyacrylamide gel and silver staining was done to resolve and observe different alleles. The observed and expected heterozygosity (H_obs_ and H_exp_) as well as forensic and paternity indices including matching probability (MP), power of discrimination (PD), power of exclusion (PE) and polymorphism information content (PIC) were calculated using the Power Stats v.1.2 software for TPOX locus of the studied population. The POPGENE (v.32) statistical package was used for analyzing allele frequency.

## Results

The 6 to 10 different alleles were detected in the studied populations with 8 allele repeats are most common in all four groups of sub-population. The maximum expected heterozygosity was found in Mansoori population (H_exp_ = 0.867). The polymorphism information content (PIC), has shown that this marker is highly informative for Mansoori (0.67) and Ansari (0.63) population group.

## Conclusion

In conclusion the interpopulation differentiation has been found significantly to differ from each other (F_ST_ = 16.37%). A dendrogram was constructed using the Neighbor-joining (NJ) clustering method. The dendrogram shows the low genetic distance between Pathan and Ansari population groups. According to the statistical parameters, the combined analysis of this TPOX STRs locus is a powerful tool for forensic personal identification, paternity testing and population genetic studies in North Indian Muslim Populations.

